# Resveratrol modulates triosephosphate isomerase and mineralization in osteosarcoma cells: potential target for novel therapeutic strategies

**DOI:** 10.55730/1300-0152.2737

**Published:** 2024-12-24

**Authors:** Gonca TUNA, Sibel ÇINAR ASA, Elif ERTÜRK, Yaren YILDIZ, Ferda ARI

**Affiliations:** 1 Department of Biology, Faculty of Arts and Sciences, Bursa Uludağ University, Bursa, Turkiye; 2 Vocational School of Health Services, Bursa Uludağ University, Bursa, Turkiye

**Keywords:** Resveratrol, osteosarcoma, triosephosphate isomerase, methylglyoxal, glycolysis, osteogenic differentiation

## Abstract

**Background/aim:**

Osteosarcoma, a primary malignant bone tumor, is challenging to treat due to its aggressive nature and limited therapies. Resveratrol (RES), a natural polyphenol, has potential anticancer properties. Hence, we investigated RES’s impact on osteosarcoma cells, focusing on triosephosphate isomerase (TPI) and related mechanisms.

**Materials and methods:**

RES was applied to osteosarcoma (SaOS-2) and healthy fetal osteoblast (hFOB 1.19) cells for 48 h, and cell viability was measured by SRB assay. The mode of cell death was examined using Hoechst 33342/annexin V/propidium iodide. TPI and methylglyoxal (MG) enzyme levels were determined by ELISA. The effect of RES on the mineralization mechanism was investigated using the Alizarin Red-S method.

**Results:**

Viability assays showed that RES significantly reduced SaOS-2 cell viability, while sparing hFOB 1.19 cells. RES treatment decreased TPI levels in SaOS-2 cells and induced MG accumulation, correlating with reduced TPI. RES also triggered apoptosis and reduced mineralization in osteosarcoma cells, affecting osteogenic differentiation.

**Conclusion:**

RES shows potential as a therapeutic agent targeting the glycolytic metabolism and apoptotic pathways in osteosarcoma cells and warrants further investigation for osteosarcoma treatment.

## Graphical Abstract

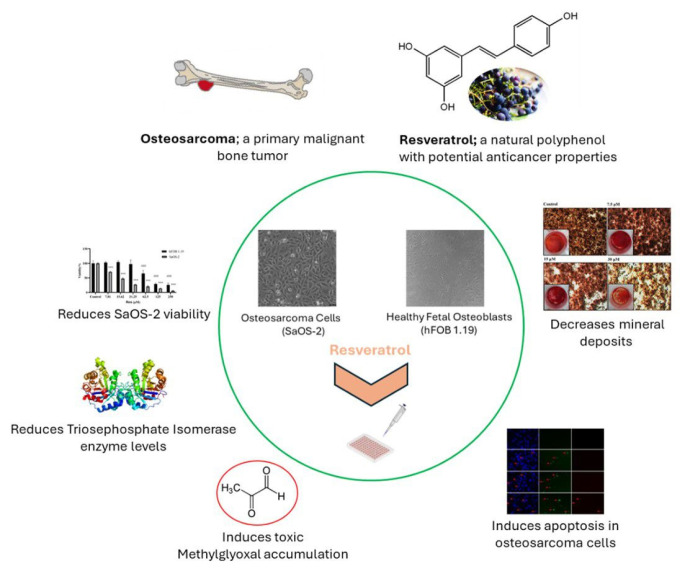

## 1. Introduction

**Figure 1:**
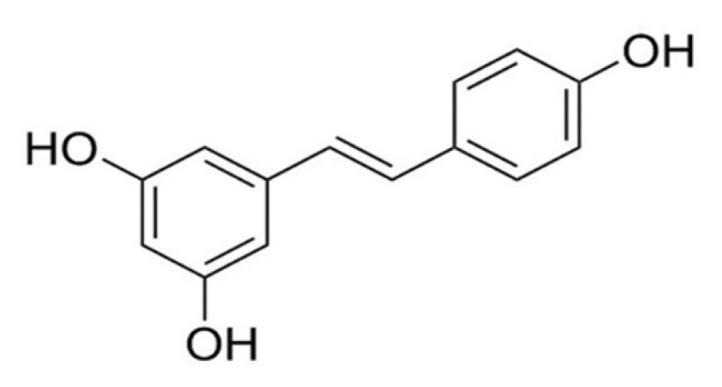
Structure of resveratrol (Camont et al., 2009).

Osteosarcoma originating from primitive mesenchymal cells is the most common type of malignant bone tumor. It originates in bone and rarely in soft tissue. Osteosarcoma can occur in any bone, but the vast majority of cases originate from the long bones of the extremities, particularly the distal femur (30%), followed by the proximal tibia (15%) and proximal humerus (15%). Left untreated, local and often metastatic progression occurs. Before polychemotherapy is administered, >90% of osteosarcoma patients die from pulmonary metastases (Fletcher et al., 2002). It is the most common primary sarcoma of bone in children and young adults but also occurs predominantly in the second decade of life and later. There are different types of osteosarcomas associated with varying degrees of aggressiveness. Generally, they are treated with chemotherapy and treatment strategies for them are similar (Moore and Luu, 2014). One of the most important characteristic features of cancer during its formation, progression, and spread is energy metabolism. As in other types of cancer, osteosarcoma cells reprogram their metabolism for rapid growth and proliferation. One of the most common metabolic changes is aerobic glycolysis, known as the Warburg effect, which results in increased glucose uptake and lactate production (Warburg, 1930, 1956; Hanahan and Weinberg, 2011). Cancer cells can perform this rearrangement of glucose metabolism by changing the levels of various glycolytic pathway enzymes (De Berardinis et al., 2008; Jones and Thompson, 2009; Granchi et al., 2014). Triosephosphate isomerase (TPI) is a key enzyme that maintains glycolysis. It catalyzes the conversion between dihydroxyacetone phosphate (DHAP) and glyceraldehydes-3-phosphate (G3P) (Solem et al., 2008). Various researchers have reported that TPI is overexpressed in various cancer types such as lung adenocarcinoma, bladder squamous cell carcinoma, and breast carcinoma (Montgomerie et al., 1997; Chen et al., 2002; Tamesa et al., 2009). In addition, overexpression of TPI has been reported in brain, lymph node, prostate, skin, kidney, and stomach cancers (Altenberg and Greulich, 2004). In particular, detailed studies have been conducted on the role of TPI in gastric cancer, and it has been found that suppression of TPI reduces cell proliferation, migration, and invasion in the gastric cancer cell line (Chen et al., 2017a, 2017b). Unlike the inhibition of other glycolytic enzymes, inhibition of TPI causes the formation of methylglyoxal (MG), a toxic intermediate. MG is a protein glycation agent and causes the formation of advanced glycation end products (AGEs) (Kold-Christensen and Johannsen, 2020). The inhibition of TPI and subsequent accumulation of MG in cancer cells could potentially impact the viability of these cells (Pekel and Arı, 2020). Studying the functions of glycolysis enzymes, signaling pathways, and both synthetic agents and natural compounds that target these pathways in anticancer drug research is crucial for developing metabolic targeted therapy strategies. One of the most researched natural products in this field is resveratrol (RES). RES is a polyphenolic compound produced in various plant species and can be found in high amounts mainly in grapes, mulberries, and peanuts (Catagol et al., 2012). The presence of attached rings in the molecule, which is a combination of a phenolic ring and a phytoalexin, allows antioxidant activity, and this structure has been shown to have the ability to scavenge hydroxyl radicals from the environment (Catagol et al., 2012; Rotches-Ribalta et al., 2012) (Figure 1). RES is known to have protective effects against cardiovascular diseases as well as antioxidant, antibacterial, antiinflammatory, and anticancer activity and neuroprotective properties (Neves et al., 2016).

The importance of RES in cancer research is that it acts as a multiple-target agent. It can affect tumor formation and progression, as well as show chemopreventive effects (Jang et al., 1997; Brockmueller et al., 2021). Specifically, it acts as a multitargeting agent by modulating signal transduction pathways that affect cell proliferation, cell cycle, apoptosis, metastasis, and angiogenesis in various types of cancer. For example, studies on osteosarcoma cells have shown that RES has a cytotoxic effect through NF-κB inhibition and could prevent cell invasion and induce apoptosis (Xu et al., 2021). It can also target multiple metabolic enzymes and metabolic signaling pathways, thus playing a role as a potential antiglycolytic agent in cancer (Kueck et al., 2007; Iqbal and Bamezai, 2012; Gomez et al., 2013). RES is also known to regulate some proteins and enzymes involved in glucose metabolism, such as the glucose transporter (GLUT), phosphofructokinase 1 (PFK1), hexokinase 2 (HK2), phosphoglycerate mutase (PGAM), glucose 6 phosphate dehydrogenase (G6PD), and transketolase (TKT) (Saunier et al., 2017). However, the effect of this compound on TPI, one of the enzymes involved in glucose metabolism and mineralization of the osteosarcoma cells, is unknown in osteosarcoma. Considering the current importance of the subject, in the present article, the effect of RES on osteosarcoma metabolism and its relationship with TPI will be discussed based on its glycolytic agent feature.

## 2. Materials and methods

### 2.1. Cell culture, osteogenic differentiation, and chemicals

Osteosarcoma cells (SaOS-2) were grown in Dulbecco’s modified Eagle’s medium (DMEM) containing 10% fetal bovine serum (FBS), 1% penicillin-G (100 U/mL)–streptomycin (100 μg/mL), L-glutamine, and sodium pyruvate. Nonmalignant fetal osteoblast cells (hFOB 1.19) were grown in DMEM/F12 (Phenol red free) containing 10% FBS, 1% penicillin-G (100 U/mL)–streptomycin (100 μg/mL), and G-418 (0.3 mg/mL). Cells were cultured at 37 °C and in 5% CO_2_.

Bone differentiation of SaOS-2 cells was performed in osteogenic differentiation medium. It consists of DMEM (D6429, Sigma-Aldrich) containing 10% FBS, 1% penicillin-G (100 U/mL)–streptomycin (100 μg/mL), 50 μg/mL ascorbic acid (A7506, Sigma-Aldrich), 10 mM β-glycerophosphate (G-9422, Sigma-Aldrich), 10^−8^ M dexamethasone (D4902, Sigma-Aldrich), L-glutamine, and sodium pyruvate in the osteogenic differentiation medium. The cells were cultured in differentiation medium containing 5% CO_2_ at 37 °C for 14 days in differentiation medium. The differentiation medium was changed every 3 days (Abdelmagid et al., 2008).

### 2.2. Sulforhodamine B (SRB) viability analysis

The SRB method is a colorimetric method based on the measurement of total protein in cells, used for the determination of cytotoxicity in cell cultures. For the SRB assay, SaOS-2 and hFOB 1.19 cells were seeded at 5 × 10^3^ cells/well in 96-well cell culture dishes. After a stock solution of RES at a concentration of 50 mM was prepared, serial dilutions ranging from 250 to 7.81 μM were performed and applied to the cells. Since the solubility of RES in water is limited, it was dissolved in DMSO. However, to minimize the potential toxic effects of DMSO, the final concentration prepared in the solution was 0.1%. After the incubation period of 48 h, the SRB method was applied as described in our previous study (Coskun et al., 2017).

### 2.3. Determination of TPI levels in osteosarcoma cells

After treatment of 7.5, 15, and 30 μM RES for 48 h, SaOS-2 and hFOB 1.19 cells were collected and cell lysates were obtained. Protein concentration in the cell lysates was determined by the bicinchoninic acid method. The protein amount in the samples was diluted to 1 μg/μL and TPI levels were determined according to the kit protocol (MyBioSource, MBS7204977).

### 2.4. Determination of MG levels

MG levels in SaOS-2 cells with and without RES treatment at 7.5, 15, and 30 μM doses were analyzed according to the protocol with the MG kit protocol (BT Lab, E4106hu). Samples were pipetted into 96-well ELISA plates coated with MG antibody. Then the MG antibody with biotin included in the kit was added followed by incubation for 1 h at 37 °C to ensure binding with the MG in the samples. Streptavidin-HRP was then added and the mixture allowed to incubate again. After 1 h of incubation, the color change that occurred by adding stop solution was determined by measuring absorbance at 450 nm.

### 2.5. Triple fluorescent staining analysis (Hoechst 33342, propidium iodide (PI), and annexin-V)

After RES application, the phenotypic characteristics of cell death in SaOS-2 cells were investigated by fluorescent staining. Hoechst, PI, and annexin-V staining were performed for apoptosis/necrosis. PI can only pass through damaged membranes, labeling only primary necrotic and late apoptotic (secondary necrosis) cells. Annexin-V stain is used to detect early apoptosis. Hoechst 33342 is a cell-permeable dye that can bind to DNA and is used to stain nuclei of live or dead (apoptotic/necrotic) cells. RES was administered at a dose of 7.5, 15, and 30 μM in SaOS-2 cells. At the end of the 48 h incubation period, cells were stained with a working solution of 3 μg/mL Hoechst 33342, 1 μg/mL PI, and 3 μg/mL annexin-V dyes. Cells were evaluated under a fluorescent microscope.

### 2.6. Alizarin Red-S staining of matrix mineralization and qualitative determination of calcium deposits

SaOS-2 cells treated with RES and the nontreated control group were grown in osteogenic medium for 14 days and then fixed with 70% ethanol. Excess alcohol was then removed and incubated by adding 0.4% Alizarin Red-S. Unbound excess dye was removed and then incubated with PBS for 15 min. Next, dehydration was carried out with 70% ethanol and then 100% ethanol. After the dyeing process was completed, extraction was performed with 10% (w/v) cetylpyridinium chloride. Supernatant absorbances were read at 540 nm with a microplate reader.

### 2.7. Statistical analysis

All statistical analyses were performed using the software package GraphPad 9.0. Percent vitality values were calculated using one-way analysis of variance (ANOVA), and if the significance was different, the difference groups were determined with Tukey’s honest significant difference (HSD) test. The results of all analyses performed with 3 replicates were given with the mean and standard deviation. Statistically significant data were determined according to p < 0.05.

## 3. Results

### 3.1. Cytotoxic effect of RES on osteosarcoma and nonmalignant fetal osteoblast cells

**Figure 2:**
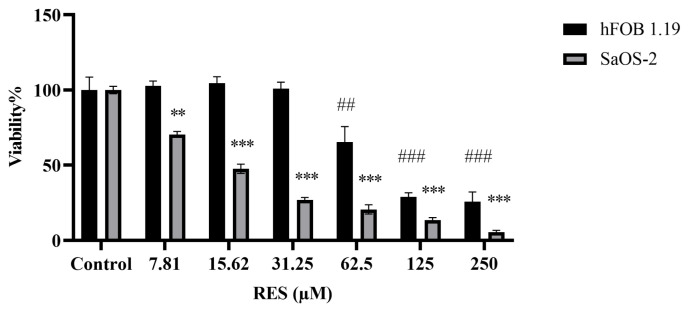
Viability of SaOS-2 and hFOB 1.19 cells treated with resveratrol (RES) for 48 h. Cells were seeded at 5 × 10^3^ cells/well in a 96-well plate. SRB assays were performed as cell viability assays. * and # indicate statistically significant differences compared to untreated control groups: ** and ## (p < 0.01), *** and ### (p < 0.001). All experiments were triplicated, and the data are presented as mean ± SD (n = 3).

After 48 h of RES administration (7.81–250 μM) to SaOS-2 and hFOB 1.19 cells, the cytotoxic effect of the compound was evaluated. It was observed that 62.5, 125, and 250 μM concentrations of RES decreased cell viability in both cell lines. However, RES at doses of 31.25 μM and lower was found to significantly reduce viability in the osteosarcoma cell line (SaOS-2) while not affecting the viability of nonmalignant fetal osteoblast cells (hFOB 1.19) (Figure 2). This minimal effect on healthy cells makes RES, which has the potential to be an anticancer agent, safe in terms of toxic side effects.

The IC_50_ values calculated for 48 h treatment according to the SRB results are 14.80 μM for SaOS-2 cells and 88.81 μM for hFOB 1.19 cells (Table). RES concentrations that did not show cytotoxicity for nonmalignant cells but were toxic to SaOS-2 cells were used for further analysis.

### 3.2. Effect of RES on TPI levels in osteosarcoma cells

**Figure 3:**
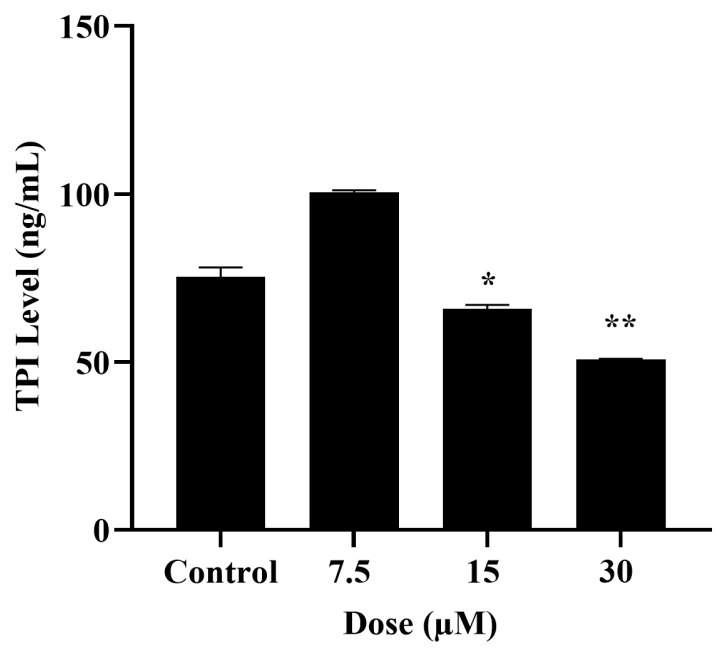
Changes in TPI levels according to ELISA results after 48 h of resveratrol (RES) application in SaOS-2 cells. Cells were seeded at 1.5 × 10^5^ cells/well in a 6-well plate and then treated with RES. All experiments were triplicated; *p < 0.05 and **p < 0.01 values indicate the statistical significance of the results compared to control groups via one-way ANOVA.

Concentrations of 7.5, 15, and 30 μM RES were applied, which caused an antiproliferative effect for SaOS-2 cells but did not cause any toxicity in hFOB 1.19 cells. Differences in TPI levels as a result of RES application were detected by the ELISA in cancer cells. It was observed that 15 and 30 μM doses of RES caused a significant decrease in TPI levels in SaOS-2 cells compared to the control cells (p < 0.05, p < 0.01) (Figure 3).

### 3.3. Effect of RES on MG formation in osteosarcoma cells

**Figure 4:**
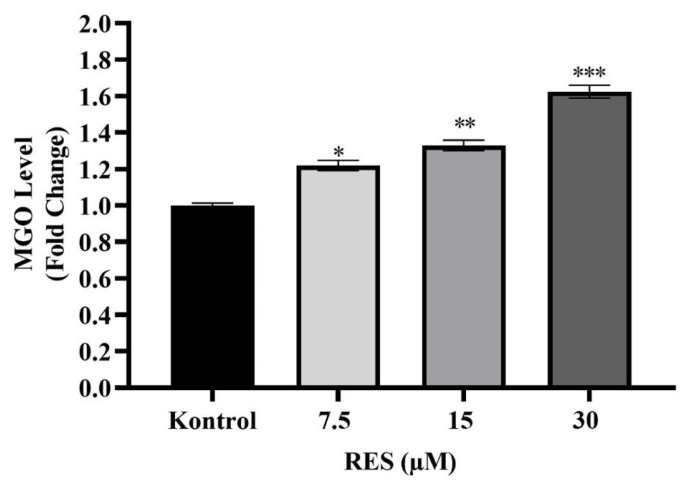
Change in methylglyoxal (MG) levels according to ELISA results after 48 h of resveratrol (RES) application in SaOS-2 cells. Cells were seeded at 1.5 × 10^5^ cells/well in a 6-well plate and then treated with RES. All experiments were triplicated; *p < 0.05, **p < 0.01, and ***p < 0.001 values indicate the statistical significance of the results compared to control groups via one-way ANOVA.

TPI is an enzyme that catalyzes the conversion between DHAP and G3P, and when this conversion does not occur, DHAP spontaneously turns into MG, a toxic intermediate. MG causes the formation of toxic and advanced glycation end products (AGEs). Therefore, defects in TPI activity may lead to the formation of MG. To examine the effect of RES on MG levels along with the decrease in TPI levels, MG formation in SaOS-2 cells was analyzed following RES application at doses of 7.5, 15, and 30 μM. A significant increase in MG levels was observed in a dose-dependent manner compared to the control (Figure 4). MG accumulation may have increased due to the decrease in TPI levels after RES application in SaOS-2 cells.

### 3.4. Fluorescent imaging results to determine the mode of cell death after RES treatment

**Figure 5:**
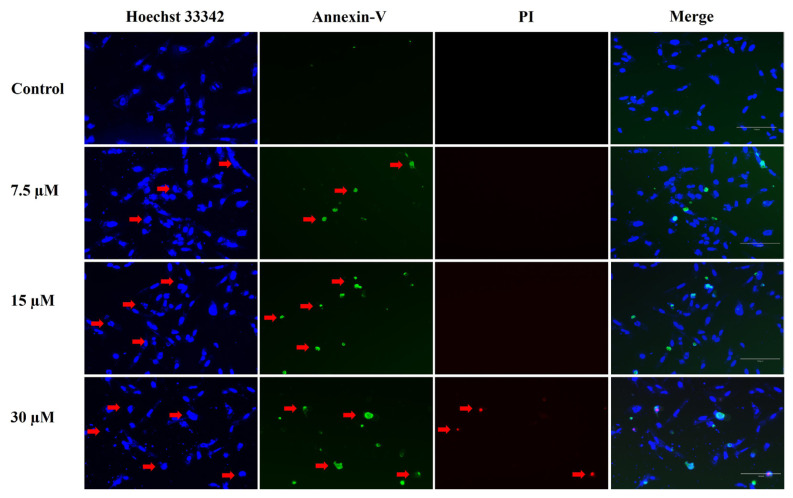
Determination of cell death mode using fluorescent imaging. SaOS-2 cells were treated with 7.5–30 μM resveratrol (RES) for 24 h. Cells were seeded at 5 × 10^3^ cells/well in a 96-well plate and then treated with RES. Cells stained red show PI staining, cells stained green show annexin staining, and cells stained blue show Hoechst 33342 staining. The merged image shows the overlaid image of Hoechst, annexin V, and PI.

Morphological changes were observed in osteosarcoma cells using the PI, annexin-V, and Hoechst 33342 triple staining method to investigate the cell death mode. SaOS-2 cells were treated with RES in the concentration range of 7.5 to 30 μM. The fluorescence imaging results of SaOS-2 cells after 24 h of RES treatment are shown in Figure 5. Fluorescence staining for the assessment of apoptotic markers was conducted after 24 h of RES treatment. This time point was chosen considering the potential increase in cytotoxic effects over time and the fact that the doses used included IC_50_ levels and above. A 48 h treatment duration results in the inability to detect apoptosis markers in fluorescent staining due to the high rate of cell death. Therefore, a 24 h treatment protocol was used to better observe the phases of apoptosis and the specific effects of the doses applied.

It is observed that the number of annexin-positive cells increases with increasing doses of RES, starting from the 7.5 μM concentration. The presence of annexin-positive cells indicates that RES treatment induces apoptosis in cells. PI negative staining of cells treated with 7.5 μM and 15 μM RES indicates that the cells are in the early stages of apoptosis. However, SaOS-2 cells showed PI positivity after the 30 μM dose of RES treatment. The increase in PI-positive cells at the 30 μM dose indicates that the RES-treated cells are in advanced stages of apoptosis (secondary necrosis/late apoptosis).

### 3.5. Effect of RES on late-stage mineral formation in osteosarcoma cells

**Figure 6:**
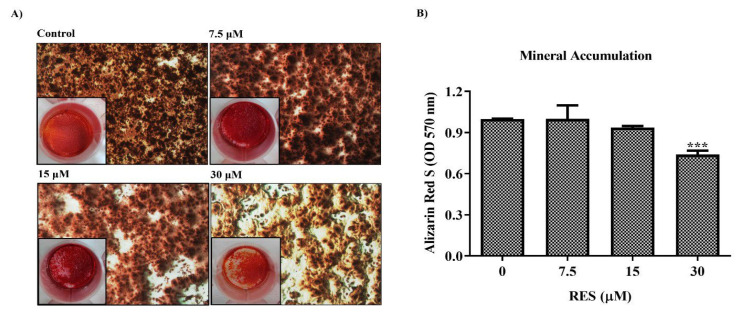
Images of SaOS-2 cells determined by Alizarin Red S staining of 14-day osteogenic differentiation after 48 h of resveratrol (RES) treatment (7.5–30 μM) (A) and quantitative determination of calcium deposits (B). Cells were seeded at 5 × 10^4^ cells/well in a 24-well plate and then treated with RES. All experiments were triplicated and *** indicates p < 0.001.

Osteosarcoma is a type of high-grade malignant bone cancer that begins in the bone-forming cells and where cancerous cells produce osteoid (bone). Therefore, changes in ossification metabolism determine whether the cell will form bone or tumor (Hofstaetter et al., 2013). Based on this information, the effects of RES on mineralization were investigated. SaOS-2 cells were treated with RES in the dose range of 7.5–30 μM for 48 h. Treated and untreated SaOS-2 cells were incubated in osteogenic differentiation medium for 14 days. Following incubation, the cells were stained with Alizarin Red-S to visualize mineralized nodule formations (Figure 6A). Alizarin Red-S is a dye used to determine calcium deposits in histology, giving an orange-to-red color when bound to calcium (Bernar et al., 2023).

To calculate the quantitative calcium value, after the staining process was completed, minerals were extracted and supernatant absorbance was measured. As a result of these analyses, it was observed that as the dose of RES increased, there was a decrease in mineralization in osteosarcoma cells compared to the control group. The decrease in Alizarin Red S dye intensity, especially at 15 and 30 μM doses, indicates that RES slows down or prevents the formation of calcium deposits. In particular, a significant decrease in mineralization was detected in the 30 μM RES-treated cells compared to the control group (Figure 6B).

## 4. Discussion

Osteosarcoma originating from mesenchymal tissue is the most common type of malignant bone tumor. As in other types of cancer, changes in glycolysis and energy metabolism in osteosarcoma are one of the main regulations for cancer cells to continue to grow and spread. TPI is a key enzyme involved in glycolysis, and it catalyzes the fifth step of glycolysis, leading to the conversion of DHAP to G3P (Maquat et al., 1985). Although the importance of TPI in cancer progression has been proven, there is very limited information about its role in osteosarcoma. However, elucidating the unknown points of metabolic changes in cancer cells is critical in cancer progression, diagnosis, and treatment (Hsu and Sabatini, 2008). Considering the current importance of natural compounds in the cancer field, in the present study, the role of TPI in the energy metabolism of osteosarcoma cells treated with RES was investigated for the first time at the molecular level. In addition, the role of RES in ossification metabolism was evaluated in the late stage of differentiation. The results showed that RES exhibited potent growth inhibitory effects in osteosarcoma cells (SaOS-2), unlike nonmalignant fetal osteoblast cells (hFOB 1.19) (Figure 2). To date, the anticancer activities of RES have been demonstrated in osteosarcomas and different cancer types. In a study that investigated the effects of RES administration on the survival of prostate carcinoma, RES was found to effectively reduce the viability of LNCaP cells depending on concentration and time (Lee and Lee, 2021). In a different study, the effect of various concentrations of RES and its esters on the viability of HepG2, A431, MCF7, HT-29, and AGS cells was determined using the MTT assay. RES at 5 μg/mL could reduce cell viability in all tested cancer cell lines except A431 cells, while RES propionate at 5 μg/mL showed cytotoxicity in all cancer cells (Oh et al., 2019). Similarly, RES treatment in osteosarcoma cells has been shown to inhibit tumor growth depending on dose and time (De Luca et al., 2022). Additionally, in a recent study on osteosarcoma, it was reported that RES applied in liposomal form to osteosarcoma cells exhibited antigrowth effects (Zhu et al., 2023). After proving the cytotoxic effect of RES on the osteosarcoma cell line, we examined its effect on TPI. TPI upregulation has been shown in different types of cancer such as stomach, brain, lymph node, prostate, kidney, testicular, colorectal, and breast cancer (Altenberg and Greulich, 2004). Misregulation of this enzyme in cancer is a known fact, but the relationship between cancer and TPI needs to be elucidated at the molecular level. Our results showed that TPI levels decreased in SaOS-2 cells after RES treatment, especially at doses of 15–30 μM (Figure 3). These results are the first showing the inhibitory effect of RES on TPI in osteosarcoma. The decrease in TPI levels suggests that it may disrupt the glycolytic pathway in osteosarcoma cells. This shows that RES has the potential to interfere with cancer metabolism by diminishing TPI levels, resulting in a reduction in glucose uptake and lactate production, indicative of a modulation in aerobic glycolysis. Several studies in the literature corroborate our findings, demonstrating the antiglycolytic activity of RES in other cancer types. Jung et al. (2013) showed that RES reduces Glut1-mediated glucose uptake by directly inhibiting PFK activity and, therefore, reduces the viability of breast cancer cells by disrupting glucose metabolism. Another study showed that RES induces autophagy in A549 lung cancer cells by upregulating glucosylceramidase beta1 (GBA1), the gene associated with Gaucher disease that codes for glucocerebrosidase, which metabolizes glucosylceramide to ceramide and glucose (Dasari et al., 2017). In colorectal cancer cells, RES modulates the lipidomic activity profile, increases oxidative activity, reduces glycolysis, and reduces pentose phosphate activity. Thus, it reverses the Warburg effect by targeting the pyruvate dehydrogenase complex. Furthermore, RES improves the oxidative capacity of colorectal cancer cells via the CamKKB/AMPK signaling pathway and suppresses glucose metabolism and tumor growth in vitro/in vivo (Jung et al., 2013; Saunier et al., 2017). In addition, it has been shown that RES reduces PFK activity in breast cancer cell lines, thereby impairing glucose metabolism and reducing cancer cell viability (Gomez et al., 2013). To better elucidate the effect of the decrease in TPI levels on osteosarcoma cells, we next examined MG levels after RES treatment. MG is a highly reactive compound with the formula CH_3_C(O)CHO (Racker, 1951). In all mammalian cells, MG is degraded by the glyoxalase system, which consists of two enzymes called glyoxalase 1 (GLO1) and glyoxalase 2 (GLO2), which catalyze the conversion of MG to d-lactate (Ray and Ray, 1998). Therefore, the effective potency of MG is expected to be greatly reduced before reaching the target malignant cells. GLO1 amplification and overexpression have been associated with cancer progression due to the degradation of MG. GLO I inhibitors began to be investigated soon after the anticancer effect of MG was reported (Szent-Györgyi, 1968). A recent study demonstrated the inhibitory effects of indomethacin against the human osteosarcoma cell line (Mirshahidi et al., 2020). Moreover, RES is known to cause cytotoxicity in breast cancer by reducing GLO1 activity (Schmidt et al., 2020). In our study, we have shown for the first time that RES treatment increased MG levels in a dose-dependent manner in osteosarcoma cells (Figure 4). This suggests that the cytotoxic effect of RES on SaOS-2 cells may be due to increased MG accumulation. Furthermore, the decreased levels of TPI following RES treatment impede its ability to catalyze the conversion between DHAP and G3P. This, in turn, results in the transformation of DHAP into MG, a toxic intermediate (Palsamy and Subramanian, 2011; Schmidt et al., 2020; Rabbani and Thornalley, 2022). As seen in the viability results, increased accumulation of toxic MG may lead to a dose-dependent increase in cytotoxicity in cells. Thus, a two-way accumulation of MG occurs, which is toxic to cancer cells. Chan et al. (2007) showed that MG treatment triggered apoptosis in human osteoblasts. Moreover, MG-induced apoptosis in osteoblasts has been shown to involve specific apoptotic biochemical changes, including oxidative stress, c-Jun N-terminal kinase (JNK) activation, mitochondrial membrane potential changes, cytochrome C release, and increased Bax/Bcl-2 proteins (Chan et al., 2007). Our results also indicate that the RES effect, in association with TPI inhibition and MG formation, can induce apoptosis in osteosarcoma cells (Figure 5). After RES treatment, it is observed that there is a significant increase in the number of annexin-positive cells with increasing doses starting from the 7.5 μM dose. Since PI-positive staining was not observed in cells treated with 7.5 and 15 μM RES, it can be concluded that they are in the early stages of apoptosis. As a result of 30 μM RES treatment, both annexin and PI-positive staining were observed in SaOS-2 cells. PI-positive staining of cells indicates that they are in the late (late apoptosis/secondary necrosis) stage of apoptosis. As a result of Hoechst 33342 staining, pycnotic and fragmented nuclei show that the cells are in apoptosis (Figure 5). Previous studies revealed that RES induces phosphatidylserine externalization in MOLT-4 cells (T lymphoblast cell line). This effect represented approximately 21% of early apoptotic cells (annexin V+/PI−) and late apoptotic/necrotic cells (annexin V+/PI+) after 24 h of RES treatment (Siedlecka-Kroplewska et al., 2019). RES was also shown to inhibit osteosarcoma proliferation and glycolysis, induce apoptosis, and reduce invasion of U2-OS cells in vitro (Xie et al., 2017). It was also observed that the apoptosis-inducing effect of RES is dose-dependent (Li et al., 2009). Similarly, in other studies conducted on many cancer types, it has been reported that RES has antiproliferative and cytotoxic effects by reducing glucose uptake and inducing apoptosis (Kueck et al., 2007; Graham et al., 2016; Li et al., 2016). Moreover, it has been reported that RES can inhibit tumor formation and induce apoptosis via JAK2/STAT3 inhibition in osteosarcoma stem cells, thus having significant anticancer potential (Peng and Jiang, 2018). We think that RES treatment may affect the glucose metabolism of the cells by causing TPI inhibition and a cytotoxic effect via MG accumulation in osteosarcoma cells. Finally, we examined the effect of RES on mineral formation to evaluate ossification in osteosarcoma cells. Mineralization refers to the process by which minerals, particularly calcium and phosphate, are deposited in the bone matrix to give it hardness and strength (Murshed, 2018). This process is essential for maintaining skeletal integrity and function. In osteosarcoma, disruption of normal bone mineralization can occur due to the presence of malignant osteoid tissue leading to the formation of structurally abnormal bone. RES has been studied for its potential to enhance mineralization in healthy bone cells by promoting osteogenic (bone-forming) activity in obese men (Ornstrup et al., 2014). However, its impact on mineralization in osteosarcoma cells, which have disrupted bone metabolism due to their malignant nature, is less understood. Our results showed a decrease in calcium deposits in SaOS-2 cells after RES treatment (Figure 6). In addition, dysregulated mineralization in osteosarcoma may also be related to glucose metabolism. While the direct relationship between mineralization and glucose metabolism in osteosarcoma is not fully elucidated, several interconnected factors may play a role. For example, osteosarcoma tumors, like many other tumors, can often exhibit areas of hypoxia (low oxygen levels) due to rapid growth and inadequate blood supply. Hypoxia triggers adaptive responses in tumor cells, including enhanced glucose uptake and reliance on glycolysis (the Warburg effect) for energy production (Si et al., 2020; Zhang et al., 2021). Therefore, the decrease in mineralization after RES treatment may be due to the disruption of glucose metabolism of osteosarcoma cells through TPI inhibition. Understanding how RES influences mineralization in relation to glucose metabolism in osteosarcoma cells could provide insights into its therapeutic mechanisms and help develop targeted treatment approaches.

## 5. Conclusion

The present study showed for the first time that RES treatment in osteosarcoma cells can disrupt glycolysis through TPI inhibition, cause MG accumulation, and thereby induce apoptosis in cells. Additionally, it has been demonstrated that RES acts to reduce late-stage mineralization in osteosarcoma cells. Our results indicate that RES can modulate TPI and related mechanisms in osteosarcoma and reveal a new potential target for therapeutic strategies. Further research is needed to fully understand the mechanisms of RES and its possible clinical implications in the treatment of osteosarcoma.

## Figures and Tables

**Table:** IC_50_ values of SaOS-2 and hFOB 1.19 cells treated with resveratrol according to SRB assay results. IC_50_ is defined as the dose inhibiting 50% of viability.

Cell Type	Time (h)	IC_50_(μM)
SaOS-2	48	14.80
hFOB 1.19	48	88.81

## Data Availability

The data that support the findings of this study are available from the corresponding author upon reasonable request.
